# Repeat self-harm and mental health service use after self-harm in Culturally and Linguistically Diverse communities: Insights from a data linkage study in Victoria, Australia

**DOI:** 10.1177/00048674231177237

**Published:** 2023-06-15

**Authors:** Thi Thu Le Pham, Kerry S O’Brien, Sara Liu, Katharine Gibson, Janneke Berecki-Gisolf

**Affiliations:** 1 Victorian Injury Surveillance Unit, Monash University Accident Research Centre, Monash University, Clayton, VIC, Australia; 2 School of Social Sciences, Monash University, Melbourne, VIC, Australia; 3 Department of Health, Melbourne, VIC, Australia

**Keywords:** Self-harm, outcomes, mental health, CALD, cultural backgrounds, country of birth, region of birth

## Abstract

**Purpose:**

To examine the associations between Culturally and Linguistically Diverse backgrounds (vs non-Culturally and Linguistically Diverse) and in-hospital death due to self-harm, repeat self-harm and mental health service use after self-harm.

**Method:**

A retrospective study of 42,127 self-harm hospital inpatients aged 15+ years in Victoria, Australia, from July 2008 to June 2019. Linked hospital and mental health service data were used to assess in-hospital death, repeat self-harm and mental health service use in the 12 months following index self-harm hospital admission. Logistic regression and zero-inflated negative binomial regression models were used to estimate associations between cultural background and outcomes.

**Results:**

Culturally and Linguistically Diverse people accounted for 13.3% of self-harm hospital inpatients. In-hospital death (0.8% of all patients) was negatively associated with Culturally and Linguistically Diverse background. Within 12 months, 12.9% of patients had self-harm readmission and 20.1% presented to emergency department with self-harm. Logistic regression components of zero-inflated negative binomial regression models showed no differences in the odds of (hospital-treated) self-harm reoccurrence between Culturally and Linguistically Diverse and non- Culturally and Linguistically Diverse self-harm inpatients. However, count components of models show that among those with repeat self-harm, Culturally and Linguistically Diverse people (e.g. born in Southern and Central Asia) made fewer additional hospital revisits than non-Culturally and Linguistically Diverse people. Clinical mental health service contacts following self-harm were made in 63.6% of patients, with Culturally and Linguistically Diverse people (Asian backgrounds 43.7%) less likely to make contact than the non-Culturally and Linguistically Diverse group (65.1%).

**Conclusions:**

Culturally and Linguistically Diverse and non-Culturally and Linguistically Diverse people did not differ in the likelihood of hospital-treated repeat self-harm, but among those with self-harm repetition Culturally and Linguistically Diverse people had fewer recurrences than non-Culturally and Linguistically Diverse people and utilised mental health services less following self-harm admissions.

## Introduction

Intentional self-harm (hereinafter referred to as self-harm) is associated with several adverse outcomes such as suicide and repeat self-harm (Bergen et al., 2012; Hawton et al., 2012; Karasouli et al., 2011). In the United Kingdom, 15–25% of adolescents returned to hospitals due to self-harm within 12 months of the first treatment for self-harm (Hawton et al., 2012; Hawton and Harriss, 2008).

The reoccurrence of self-harm may be influenced by cultural factors. In the United Kingdom, White study participants were more likely to report repeated incidents of self-harm (Borrill et al., 2011) than ethnic minority groups. Studies in England and Australia (Li et al., 1999; Wynaden et al., 2005) reported that uptake of mental health services after self-harm, which has the potential to prevent suicide and repeat self-harm (Carter et al., 2016; Spittal et al., 2017), is affected by cultural beliefs, stigma and shame.

However, past studies on self-harm among Culturally and Linguistically Diverse (CALD) populations have primarily focused on European countries with few studies on Australia (Borrill et al., 2011; Cooper et al., 2006), despite Australia being one of the most multicultural migrant countries, and well-suited for studying self-harm outcomes in CALD people. Therefore, we aimed to address this gap by examining outcomes of hospital-admitted self-harm among people from CALD and non-CALD communities. Specifically, we sought to examine the associations between cultural backgrounds (regions of birth [ROBs]) and in-hospital death, repeated self-harm and mental health services use after self-harm. Because people from CALD backgrounds might report self-harm as an unintentional injury (El Halabi et al., 2020) due to shame and stigma, we also tested the relationship between CALD backgrounds and subsequent hospital treatment due to injury.

## Methodology

This population-based cross-sectional study was conducted using linked hospital and mental health data in Victoria, Australia. We followed self-harm hospital inpatients (sourced from the Victorian Admitted Episodes Dataset – VAED) in linked data of VAED, Victorian Emergency Minimum Dataset – VEMD, and clinical public mental health service use data – CMI/ODS to identify outcomes following self-harm hospital admissions in different CALD groups versus non-CALD.

The linkage process was conducted by the Centre for Victorian Data Linkage using a unique record number supplied to the researchers in de-identified format. Identifiers were removed and dates were replaced with encrypted dates; therefore, informed consent from participants was not needed. The data sources and data linkage process are explained in Appendix 1.

### Participants

In the VAED, self-harm admissions were extracted by selecting admission records containing a first external cause (ICD-10-AM–the International Classification of Diseases and Related Health Problems, Tenth Revision, Australian Modifications) indicating self-harm (X60–X84), and a principal diagnosis that was either an injury (S00–T98) or a mental or behavioural disorder (F00–F99) (Clapperton, 2019). *Self-harm events* captured in this study were episodes of self-harm with or without the intended death.

From the VAED, self-harm hospital admission episodes were first grouped into periods of care (POC) by identifying the incident episode and subsequent transfers and statistical separations within less than 2 days between separation and admission. Then we selected patients with an index (initial) self-harm POC admission occurring between 01 July 2008 and 30 June 2019. Note that patients may have engaged in self-harm previously to the timeframe examined here. We did not exclude those with self-harm admission that occurred before 01 July 2008. But we did record those who had at least one self-harm admission 12 months before the index self-harm event as pre-existing self-harm admissions (including pre-existing conditions that occurred within 12 months before 01 July 2008).

Only male or female patients aged 15 years and above living in Victoria (regardless of their visa status) were included. Sex other than male or female was rare, and these small cell counts could compromise data confidentiality. We excluded observations if patients had missing information on their country of birth in the VAED (<1.5% of otherwise eligible cases).

**Figure 1.**
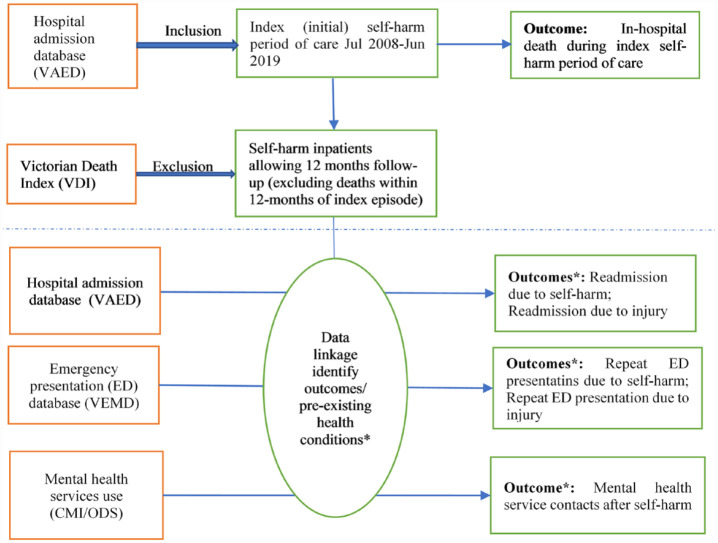
Flow diagram to outline the linkage methodology of the study. VAED: Victorian Admitted Episodes Dataset; VEMD: Victorian Emergency Minimum Dataset; CMI/ODS: Clinical Client Management Interface/Operational Data Store; VDI: Victorian Death Index. *Previous existing health conditions were identified by 12 months health services use look back (2007/2008–2018/2019).

To assess in-hospital death, all individuals in the sample selected above were included. However, to evaluate re-admission/presentation and mental health service use outcomes, we only included those that allow the analysis of 12 months follow-up. This means those who died in hospital during the index POC (as recorded in the VAED), and those who died within 12 months after index admission (as recorded in the Victorian Death Index) were removed when examining the 12 months health service use outcomes (Figure 1).

### Outcomes

Six outcomes were identified.

1. In-hospital death during the index POCs: a binary outcome from VAED;
2. Self-harm readmission: coded as an index admission or repeated admission based on the time of occurrence. Repeated self-harm/injury admission was counted as unplanned admissions within 12 months of the index separation date from linked VAED;
3. Repeat self-harm emergency department (ED) presentation: coded as presentations due to self-harm within 12 months of the index separation date (VEMD linked with VAED);
4. and 5. Similarly, readmission/repeat ED presentations due to injury (codes S00-T79);
6. Mental health service utilisation: occurred within 12 months of the self-harm index separation date (CMI-ODS linked with VAED).

### CALD variables

While combining all CALD people into a CALD group is common in current practice and policy, studies have shown that this group is highly heterogeneous and should not be grouped into one group in self-harm research (Pham et al., 2023; Troya et al., 2022). This study conducted two separate analyses of (1) CALD as one group and (2) CALD groups divided by ROBs to have more specific results.

#### CALD/Non-CALD status of participants

CALD status was determined based on the country of birth, as recorded in the VAED. The CALD group consisted of those who were born in non-English-speaking countries (Pham et al., 2021). We did not use ‘main language spoken at home’ and ‘Indigenous status’ in the CALD selection process. ‘Language spoken at home’ was not available in the data and therefore could not be captured. The small Indigenous population size in Victoria (compared with CALD and non-CALD populations) rendered it statistically unfeasible to include this as a separate group. Although Indigenous peoples should be in a separate group for data analysis due to their cultural diversity (as well as having relatively high rates of self-harm [Australian Institute of Health and Welfare (AIHW), 2022]), the small sample size might affect the statistical power and data confidentiality of the research. For this reason, Indigenous peoples were grouped into CALD or non-CALD groups depending on their country of birth. Although this grouping has limitations as outlined above, these limitations are likely to lower the sensitivity of the analysis in being able to distinguish CALD/non-CALD differences: therefore, the findings in this paper may present an underestimate of the underlying differences in outcomes between these groups.

#### CALD groups by 9 regions of birth of participants (9 ROB/ non-CALD)

CALD participants were divided into nine ROB based on the Australian Bureau of Statistics (ABS)’s region classifications, specifically:

- Oceania and Antarctica region, excluding Australia and New Zealand*;
- North-West Europe, excluding the United Kingdom (England, Wales, Scotland, Northern Ireland) and the Republic of Ireland*;
- Southern and Eastern Europe;
- North Africa and the Middle East;
- South-East Asia;
- North-East Asia;
- Southern and Central Asia;
- Americas, excluding Canada and the United States*;
- Sub-Saharan Africa, excluding South Africa*.

* Persons who were born in English-speaking countries were excluded from the relevant CALD groups by ROB, and they were included in the non-CALD group.

**Figure 2.**
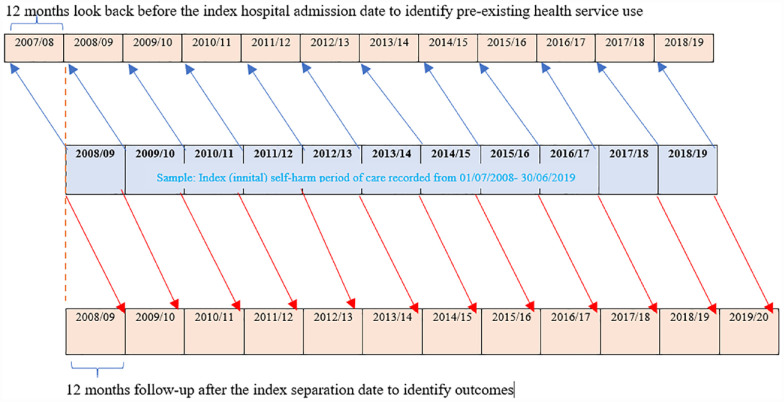
Schematic diagram of the data linkage periods to determine (a) pre-existing health service use and (b) post-event health service use (outcomes), within 12 months.

*Pre-existing health service use within 12 months before the index admission date* including *pre-existing* hospital admissions/ED presentation due to self-harm, hospital-treated injury, any mental illness admission and mental health contacts before index admission were coded (with the same approach that was used for determining outcomes of self-harm). Specifically, VAED, VEMD and CMI/ODS records from 01 July 2007 to 30 June 2019 were linked with the index self-harm hospital admission episodes and look-back for 12 months (Figures 1 and 2).

### Variables

Demographic variables including sex, age group and marital status were extracted from the VAED; remoteness (Accessibility and Remoteness Index of Australia - ARIA+) an socioeconomic status (Socio-Economic Indexes for Areas - SEIFA [ABS, 2018]) of participants were defined based on the postcode of residential address of patients.

Injury-related variables included mechanisms of self-harm, place of injury, comorbidity (Charlson et al., 1987) any psychiatric disorder admission (F00-F99), and alcohol use mentioned in index hospital admission (McKenzie et al., 2010) were coded based on ICD-10-AM codes obtained from VAED.

Injury severity was calculated using age group-stratified ICD-based injury severity scores (ICISS) based on the worst-injury method (Berecki-Gisolf et al., 2022). A serious injury was considered to be one with an ICISS of less than 0.941 (survival probability of 94.1% or less) (Cryer et al., 2008).

### Statistical methods

Data were analysed using SAS version 14.1, SPSS version 25 and STATA version 12.1. We compared frequency distributions of key variables between groups with different CALD regions of birth and the non-CALD group (Table 1). Serious injuries, which accounted for 0.9% of patients and pre-existing self-harm admission (1.5% of patients) are not presented due to confidentiality reasons (small cell counts); however, these variables were included in the modelling. To compare the outcomes of self-harm (binary variables) among self-harm admissions of people from different CALD regions of birth and the non-CALD population, Chi-square tests were performed (Table 2).

**Table 1.** Characteristics of self-harm hospital inpatients in Victoria from July 2008 to June 2019.

Characteristics	Non-CALD*n* (%)	Oceania and Antarctica* *n* (%)	North-West Europe* *n* (%)	Southern and Eastern Europe *n* (%)	North Africa and the Middle East *n* (%)	South-East Asia*n* (%)	North-East Asia *n* (%)	Southern and Central Asia *n* (%)	Americas* *n* (%)	Sub-Saharan Africa* *n* (%)
**Total**	35,461	142	500	1170	811	914	456	1022	175	241
	(86.7)	(0.3)	(1.2)	(2.9)	(2.0)	(2.2)	(1.1)	(2.5)	(0.4)	(0.6)
**Sex**										
Male	13,393	55	192	487	311	274	111	381	57	99
	(37.8)	(38.7)	(38.4)	(41.6)	(38.4)	(30.0)	(24.3)	(37.3)	(32.6)	(41.1)
Female	22,068	87	308	683	500	640	345	641	118	142
	(*62.2*)	(*61.3*)	(*61.6*)	(*58.4*)	(*61.7*)	(*70.0*)	(*75.7*)	(*62.7*)	(*67.4*)	(*58.9*)
**Age groups**										
15–24	12,351	35	62	63	205	259	169	324	34	73
	(*35.1*)	(*24.8*)	(*12.5*)	(*5.4*)	(*25.3*)	(*28.3*)	(*37.1*)	(*31.7*)	(*19.4*)	(*30.3*)
25–34	6990	27	58	102	209	257	121	404	42	63
	(*19.8*)	(*19.2*)	(*11.7*)	(*8.7*)	(*25.8*)	(*28.1*)	(*26.5*)	(*39.5*)	(*24*)	(*26.1*)
35–44	6689	54	60	151	173	206	68	166	34	46
	(*19.0*)	(*38.3*)	(*12.1*)	(*12.9*)	(*21.4*)	(*22.5*)	(*14.9*)	(*16.2*)	(*19.4*)	(*19.1*)
45–54	5191	15	79	247	119	99	43	67	40	27
	(*14.7*)	(*10.6*)	(*15.9*)	(*21.2*)	(*14.7*)	(*10.8*)	(*9.4*)	(*6.6*)	(*22.9*)	(*11.2*)
55–64	2416	‡	108	207	64	54	33	36	13	19
	(*6.9*)		(*21.7*)	(*17.7*)	(*7.9*)	(*5.9*)	(*7.2*)	(*3.5*)	(*7.4*)	(*7.9*)
65 +	1591	†	131	397	39	39	22	25	12	13
	(*4.5*)		(*26.3*)	(*34*)	(*4.8*)	(*4.3*)	(*4.8*)	(*2.5*)	(*6.9*)	(*5.4*)
**Remoteness (Accessibility and Remoteness Index of Australia - ARIA+)**										
Major Cities of Australia	20,123	110	331	893	670	736	386	803	141	189
	(*56.8*)	(*77.5*)	(*66.2*)	(*76.3*)	(*82.6*)	(*80.5*)	(*84.6*)	(*78.6*)	(*80.6*)	(*78.4*)
Inner Regional Australia	12,098	24	132	254	115	135	29	140	‡	31
	(*34.2*)	(*16.9*)	(*26.4*)	(*21.7*)	(*14.2*)	(*14.8*)	(*6.4*)	(*13.7*)		(*12.9*)
Outer Regional Australia	2838	7	20	13	7	16	5	13	†	7
	(*8.0*)	(*4.9*)	(*4.0*)	(*1.1*)	(*0.9*)	(*1.8*)	(*1.1*)	(*1.3*)		(*2.9*)
**Socio-Economic Indexes for Areas (SEIFA)**										
Decile 1–5	10,758	44	90	258	277	283	43	263	29	52
	(*30.7*)	(*31.2*)	(*18.6*)	(*22.2*)	(*35*)	(*31.9*)	(*10.2*)	(*27.5*)	(*17.1*)	(*22.9*)
Decile 6–10 (greater advantage)	24,307	97	393	902	515	604	377	693	141	175
	(*69.3*)	(*68.7*)	(*81.4*)	(*77.8*)	(*65*)	(*68.1*)	(*89.8*)	(*72.5*)	(*82.9*)	(*77.1*)
**Marital status**										
Never married	22,151	63	143	242	331	468	254	406	82	123
	(*62.5*)	(*44.4*)	(*28.6*)	(*20.7*)	(*40.8*)	(*51.2*)	(*55.7*)	(*39.7*)	(*46.9*)	(*51.0*)
Widowed/Divorced/Separated	3894	13	120	287	142	96	32	78	34	33
	(*11.0*)	(*9.2*)	(*24.0*)	(*24.5*)	(*17.5*)	(*10.5*)	(*7.0*)	(*7.6*)	(*19.4*)	(*13.7*)
Currently married/defacto	8578	65	221	628	323	328	160	525	58	80
	(*24.2*)	(*45.8*)	(*44.2*)	(*53.7*)	(*39.8*)	(*35.9*)	(*35.1*)	(*51.4*)	(*33.1*)	(*33.2*)
**Alcohol mentioned in diagnosis codes**										
Yes	9685	38	181	268	105	119	75	105	49	46
	(*27.3*)	(*26.8*)	(*36.2*)	(*22.9*)	(*13.0*)	(*13.0*)	*16.5*)	(*10.3*)	(*28.0*)	(*19.1*)
No	25,776	104	319	902	706	795	381	917	126	195
	(*72.7*)	(*73.2*)	(*63.8*)	(*77.1*)	(*87.1*)	(*87.0*)	(*83.6*)	(*89.7*)	(*72.0*)	(*80.9*)
**Mechanism of self-harm**										
Poisoning-pharmaceuticals	27,997	94	389	910	656	728	340	724	139	173
	(*79.0*)	(*66.2*)	(*77.8*)	(*77.8*)	(*80.9*)	(*79.7*)	(*74.6*)	(*70.8*)	(*79.4*)	(*71.8*)
Poisoning other substances	1698	19	30	87	46	42	24	148	9	32
	(*4.8*)	(*13.4*)	(*6.0*)	(*7.4*)	(*5.7*)	(*4.6*)	(*5.3*)	(*14.5*)	(*5.1*)	(*13.3*)
Sharp object	3985	17	51	114	77	94	70	110	18	21
	(*11.2*)	(*12.0*)	(*10.2*)	(*9.7*)	(*9.5*)	(*10.3*)	(*15.4*)	(*10.8*)	(*10.3*)	(*8.7*)
Other means	1781	12	30	59	32	50	22	40	9	15
	(*5.0*)	(*8.5*)	(*6.0*)	(*5.0*)	(*4.0*)	(*5.5*)	(*4.8*)	(*3.9*)	(*5.1*)	(*6.2*)
**Place of injury**										
Home	16,913	73	256	685	418	437	223	536	90	123
	(*47.7*)	(*51.4*)	(*51.2*)	(*58.6*)	(*51.5*)	(*47.8*)	(*48.9*)	(*52.5*)	(*51.4*)	(*51.0*)
Health care centre	2313	9	29	55	43	28	17	37	13	8
	(*6.5*)	(*6.3*)	(*5.8*)	(*4.7*)	(*5.3*)	(*3.1*)	(*3.7*)	(*3.6*)	(*7.4*)	(*3.3*)
Other settings	2283	6	35	73	48	56	23	55	14	18
	(*6.4*)	(*4.2*)	(*7.0*)	(*6.2*)	(*5.9*)	(*6.1*)	(*5.0*)	(*5.4*)	(*8.0*)	(*7.5*)
Missing	13,952	54	180	357	302	393	193	394	58	92
	(*39.3*)	(*38.0*)	(*36.0*)	(*30.5*)	(*37.2*)	(*43.0*)	(*42.3*)	(*38.6*)	(*33.1*)	(*38.2*)
**Length of stay**										
<2 days	21,688	92	246	574	509	539	269	683	109	155
	(*61.2*)	(*64.8*)	(*49.2*)	(*49.1*)	(*62.8*)	(*59.0*)	(*59.0*)	(*66.8*)	(*62.3*)	(*64.3*)
2–7 days	8359	29	126	280	199	227	109	216	35	44
	(*23.6*)	(*20.4*)	(*25.2*)	(*23.9*)	(*24.5*)	(*24.8*)	(*23.9*)	(*21.1*)	(*20.0*)	(*18.3*)
8–30 days	4136	14	95	224	85	115	58	94	21	31
	(*11.7*)	(*9.9*)	(*19.0*)	(*19.2*)	(*10.5*)	(*12.6*)	(*12.7*)	(*9.2*)	(*12.0*)	(*12.9*)
31 + days	1278	7	33	92	18	33	20	29	10	11
	(*3.6*)	(*4.9*)	(*6.6*)	(*7.9*)	(*2.2*)	(*3.6*)	(*4.4*)	(*2.8*)	(*5.7*)	(*4.6*)
**Within the index hospital admission**										
Any comorbidity CHARLSON	1913	10	44	172	57	74	24	65	7	19
	(*5.4*)	(*7.0*)	(*8.8*)	(*14.7*)	(*7.0*)	(*8.1*)	(*5.3*)	(*6.4*)	(*4.0*)	(*7.9*)
Any mental health disorder	19,065	68	309	698	394	457	215	446	88	127
	(*53.8*)	(*47.9*)	(*61.8*)	(*59.7*)	(*48.6*)	(*50.0*)	(*47.2*)	(*43.6*)	(*50.3*)	(*52.7*)
**Pre-existing service use in the last 12** months**										
Hospital admission due to injury	3158	10	43	89	63	41	16	42	11	13
	(*8.9*)	(*7.0*)	(*8.6*)	(*7.6*)	(*7.8*)	(*4.5*)	(*3.5*)	(*4.1*)	(*6.3*)	(*5.4*)
ED presentation due to self-harm	2829	8	33	44	36	35	13	23	6	†
	(*8.0*)	(*5.6*)	(*6.6*)	(*3.8*)	(*4.4*)	(*3.8*)	(*2.9*)	(*2.3*)	(*3.4*)	†
ED presentation due to injury	13,398	54	160	320	253	239	101	266	62	68
	(*37.8*)	(*38.0*)	(*32.0*)	(*27.4*)	(*31.2*)	(*26.2*)	(*22.2*)	(*26.0*)	(*35.4*)	(*28.2)*
Hospital admission due to mental health disorder (F00-F99)	8663	27	117	228	134	116	50	112	34	46
	(*24.4*)	(*19.0*)	(*23.4*)	(*19.5*)	(*16.5*)	(*12.7*)	(*11.0*)	(*11.0*)	(*19.4*)	(*19.1)*
Clinical mental health service (CMI-ODS)	11,121	30	123	258	166	143	64	117	50	53
	(*31.4*)	(*21.1*)	(*24.6*)	(*22.1*)	(*20.5*)	(*15.7*)	(*14.0*)	(*11.5*)	(*28.6*)	(*22.0)*

ED: emergency department; CMI/ODS: Clinical Client Management Interface/Operational Data Store; POCS: periods of care; CALD: Culturally and Linguistically Diverse; ROB: region of birth.

* Persons/POCs of those who were born in English-speaking countries were excluded from the relevant CALD groups by ROB, and they were included in the non-CALD group.

** Due to small count cells (confidential reasons), injury severity and pre-existing hospital admission due to ISH were not reported in the table but they were included in the modelling.

† Suppressed because cell values were less than 5.

‡ Other cells in the same row and/or column may also be suppressed to maintain confidentiality.

**Table 2.** Binary outcomes of self-harm within 12 months after the index self-harm event.

**Outcomes**	Non-CALD *n* (%)	Oceania and Antarctica* *n* (%)	North-West Europe* *n* (%)	Southern and Eastern Europe *n* (%)	North Africa and the Middle East *n* (%)	South-East Asia *n* (%)	North-East Asia*n* (%)	Southern and Central Asia *n* (%)	Americas* *n* (%)	Sub-Saharan Africa* *n* (%)
1. In-hospital death during the period of care** (*n* = 42,127 patients)	270	†	11	18	†	7	†	9	†	†
	(0,7)		(2,1)	(1,4)		(0,8)		(0,9)		
2. Subsequent admission due to self-harm (*n* = 40,892 patients)	4819	16	64	99	80	65	29	53	17	18
	(13.6)	(*11.3*)	(*12.8*)	(*8.5*)	(*9.9*)	(*7.1*)	(*6.4*)	(*5.2*)	(*9.7*)	(*7.5*)
3. Subsequent admission due to unintentional injuries (*n* = 40,892 patients)	6518	25	94	161	112	94	40	92	26	22
	(18.4)	(17.6)	(*18.8*)	(*13.8*)	(*13.8*)	(*10.3*)	(*8.8*)	(*9.0*)	(*14.9*)	(*9.1*)
4. Subsequent ED presentations due to self-harm (*n* = 40,892 patients)	7413	27	89	155	126	135	59	143	32	39
	(20.9)	(19.0)	(*17.8*)	(*13.3*)	(*15.5*)	(*14.8*)	(*12.9*)	(*14.0*)	(*18.3*)	(*16.2)*
5. Subsequent ED presentations due to unintentional injuries (*n* = 40,892 patients)	13,398	54	160	320	253	239	101	266	62	68
	(37.8)	(38.0)	(*32.0*)	(*27.4*)	(*31.2*)	(*26.2*)	(*22.2*)	(*26.0*)	(*35.4*)	(*28.2)*
6. Mental health contact after self-harm (CMI/ODS) (*n* = 40,892 patients)	23,091	85	311	754	483	477	185	382	107	137
	(65.1)	(59.9)	(*62.2*)	(*64.4*)	(*59.6*)	(*52.2*)	(*40.6*)	(*37.4*)	(*61.1*)	(*56.9)*
**Total**	**35,461**	**142**	**500**	**1170**	**811**	**914**	**456**	**1022**	**175**	**241**

CALD: Culturally and Linguistically Diverse; ED: emergency department; CMI/ODS: Clinical Client Management Interface/Operational Data Store; ROB: region of birth.

* Persons who were born in English-speaking countries were excluded from the relevant CALD groups by ROB, and they were included in the non-CALD group.

** Only with the analysis of in-hospital death outcome, deaths in hospital and within 12 months of discharge from hospital were included, these cases were excluded from all other analyses.

† Suppressed because cell values were less than 5 to maintain confidentiality.

*p* < 0.0001 (Chi-square tests) for outcomes. Due to the low incidence of in-hospital death in some CALD groups, the differences between CALD regions of birth versus non-CALD was not tested.

To evaluate the association between CALD status and the outcomes of self-harm, univariable and fully adjusted modelling (*adjusted for demographic variables, injury-related variables and pre-existing health service use*) were carried out (Table 3):

1. Count modelling was conducted for the following count outcomes: readmissions and ED presentations due to self-harm, readmission/ED presentation due to injury and mental health service contact after the index self-harm admission.

We used zero-inflated negative binomial regression (ZINB) distribution to perform count models. ZINB is a maximum-likelihood count regression analysis, designed for non-normal (i.e. skewed and over-dispersed) count data, specifically suitable for the health service use data with an excessive number of zeros observed in this study (Liu and Cela, 2008). The model consisted of two distributions to reflect two different processes that influence the occurrence of events and the frequency of events. Vuong test was conducted and it showed the ZINB model as preferred against the standard negative binomial regression models for all outcomes (Liu and Cela, 2008).

2. Logistic regression modelling was performed to test the association between CALD status (binary) and in-hospital death during the first self-harm POC. Due to the low incidence of in-hospital death in some CALD groups, the relationship between CALD regions of birth versus non-CALD was not tested.

**Table 3.** Associations between CALD status and repeat self-harm, mental health service use 1 year following index self-harm hospital admission. Reference group: Non-CALD.

Outcomes	In hospital death			Readmission due to self-harm						Repeat ED presentation due to self-harm						Mental health service use after self-harm					
	OR	*95% CI*		IRR	*95% CI*		OR	*95% CI*		IRR	*95% CI*		OR	*95% CI*		IRR	*95% CI*		OR	*95% CI*	
**Univariable models**																					
**CALD status**	**1.3** ^†^	*****1.0*****	***1.8***	**0.7** ^†^	***0.6***	***0.9***	**1.0**	***0.8***	***1.3***	**0.7** ^†^	***0.7***	***0.8***	**0.2** ^†^	***0.1***	***0.6***	**0.8** ^†^	***0.7***	***0.8***	**2.0** ^†^	***1.8***	***2.2***
**CALD groups by regions of birth**																					
Oceania and Antarctica*				0.7	*0.3*	*1.5*	1.0	*0.2*	*3.6*	0.5^†^	*0.3*	*0.9*	0.3	*0.0*	*2.7*	0.9	*0.6*	*1.2*	1.5	*0.7*	*2.9*
North-West Europe*				0.8	*0.6*	*1.1*	0.6	*0.3*	*1.2*	0.6^†^	*0.5*	*0.8*	0.2^†^	*0.1*	*0.6*	0.9	*0.8*	*1.1*	1.0	*0.6*	*1.6*
Southern and Eastern Europe*				0.7^†^	*0.5*	*0.9*	0.8	*0.5*	*1.3*	0.5^†^	*0.4*	*0.7*	0.3^†^	*0.1*	*0.6*	0.8^†^	*0.7*	*0.9*	0.9	*0.6*	*1.2*
North Africa and the Middle East				0.7^†^	*0.5*	*1.0*	0.8	*0.4*	*1.3*	0.5^†^	*0.4*	*0.7*	0.5	*0.2*	*1.6*	0.7^†^	*0.6*	*0.8*	1.4^†^	*1.0*	*1.9*
South-East Asia				0.6^†^	*0.4*	*0.9*	0.9	*0.5*	*1.7*	0.5^†^	*0.4*	*0.6*	0.5	*0.2*	*1.3*	0.7^†^	*0.6*	*0.8*	2.2^†^	*1.7*	*2.8*
North-East Asia				0.8	*0.4*	*1.3*	1.2	*0.5*	*2.6*	0.6^†^	*0.4*	*0.9*	1.3	*0.5*	*4.0*	1.0	*0.8*	*1.3*	4.1^†^	*3.1*	*5.4*
Southern and Central Asia				0.6^†^	*0.4*	*1.0*	1.4	*0.7*	*2.6*	0.4^†^	*0.3*	*0.5*	0.5	*0.2*	*1.3*	0.6^†^	*0.5*	*0.8*	4.7^†^	*3.9*	*5.7*
Americas*				2.2	*1.2*	*3.9*	3.3	*1.6*	*7.0*	1.0	*0.6*	*1.6*	1.2	*0.4*	*4.2*	0.8	*0.6*	*1.1*	1.3	*0.6*	*2.5*
Sub-Saharan Africa*				0.3^†^	*0.2*	*0.7*	0.3	*0.1*	*1.3*	0.4	*0.2*	*0.6*	0.1	*0.0*	*2.2*	1.0	*0.8*	*1.4*	1.8^†^	*1.1*	*2.8*
**Multivariable models**																					
**CALD status**	**0.7** ^†^	***0.5***	***0.9***	**0.8** ^†^	***0.7***	***0.9***	**1.1**	***0.8***	***1.7***	**0.9** ^†^	***0.8***	***0.9***	**0.7**	***0.3***	***2.0***	**0.9** ^†^	***0.9***	***1.0***	**1.4** ^†^	***1.2***	***1.5***
**CALD groups by regions of birth**																					
Oceania and Antarctica*				0.7	*0.4*	*1.3*	0.8	*0.1*	*6.8*	0.7	*0.5*	*1.1*	0.6	*0.0*	*40.1*	0.8	*0.6*	*1.1*	1.0	*0.6*	*1.7*
North-West Europe*				1.0	*0.8*	*1.4*	0.8	*0.3*	*2.5*	0.9	*0.7*	*1.2*	1.2	*0.1*	*13.5*	0.9	*0.8*	*1.1*	0.9	*0.7*	*1.2*
Southern and Eastern Europe*				1.0	*0.8*	*1.2*	1.1	*0.5*	*2.2*	1.0	*0.9*	*1.2*	1.4	*0.3*	*6.5*	0.9	*0.8*	*1.0*	0.9	*0.7*	*1.1*
North Africa and the Middle East				0.8	*0.6*	*1.1*	0.8	*0.3*	*2.2*	0.7^†^	*0.6*	*0.9*	0.2	*0.0*	*1.1*	1.0	*0.9*	*1.1*	1.0	*0.8*	*1.3*
South-East Asia				0.8^†^	*0.6*	*1.0*	2.0	*0.8*	*4.8*	0.9	*0.7*	*1.0*	1.2	*0.1*	*13.6*	0.9^†^	*0.8*	*1.0*	1.4^†^	*1.1*	*1.7*
North-East Asia				0.7	*0.5*	*1.1*	1.1	*0.3*	*3.7*	1.0	*0.7*	*1.3*	0.9	*0.0*	*18.0*	1.2^†^	*1.0*	*1.5*	2.4^†^	*1.8*	*3.1*
Southern and Central Asia				0.6^†^	*0.4*	*0.8*	1.0	*0.4*	*2.6*	0.8^†^	*0.7*	*1.0*	3.4	*0.8*	*14.4*	0.8^†^	*0.7*	*0.9*	2.3^†^	*1.9*	*2.8*
Americas*				1.4	*0.9*	*2.2*	4.0	*0.8*	*21.0*	1.1	*0.8*	*1.6*	0.7	*0.0*	*55.9*	1.1	*0.8*	*1.4*	1.2	*0.8*	*2.0*
Sub-Saharan Africa*				0.6	*0.3*	*1.1*	0.4	*0.0*	*5.0*	0.8	*0.5*	*1.1*	0.3	*0.0*	*7.8*	1.3^†^	*1.1*	*1.7*	1.2	*0.9*	*1.8*

CALD: Culturally and Linguistically Diverse; ED: emergency department; OR: odds ratio; CI: confidence interval; IRR: Incident Risk Ratio; ROB: region of birth; ZINB: zero-inflated negative binomial regression.

Due to the low incidences of in-hospital death in some CALD regions, the difference between CALD regions of birth versus non-CALD was not tested.

Please see Appendix 2 for the results of ZINB models for injury outcomes (readmission due to injury and repeat ED presentation due to injury).

* Persons who were born in English-speaking countries were excluded from the relevant CALD groups by ROB, and they were included in the non-CALD group.

† : *p* < 0.05.

### Ethical consideration

Ethical approval was provided by the Monash University Human Research Ethics Committee (Project ID 27360).

## Results

### Sample

There were 42,127 individuals hospitalised due to self-harm in Victoria between July 2008 to June 2019 and selected for the study; among them, a total of 325 died while in hospital during index POC and a further 910 died within 12 months of the index hospital discharge. All who died were excluded from the analysis of health services use after hospital discharge (but not excluded from the in-hospital death analysis), leaving a remaining sample of 40,892 patients.

#### Characteristics of self-harm in nine specific regions of birth versus the non-CALD group

There were 5434 patients with CALD backgrounds (13.3% of self-harm patients); 1170 originally from Southern and Eastern Europe (2.9%), 1022 from Southern and Central Asia (2.5%) and 914 were from South-East Asia (2.2%). Females were overrepresented in all CALD groups, but this was most pronounced among those born in Asia (e.g. North-East Asia 76%) compared to the sample overall (62%). Southern- and Eastern Europe–born patients were the oldest of all groups; in all other groups, the number of patients tended to decrease as age increased, whereas in this group the number of patients increased as age increased. Poisoning by pharmaceuticals was the most common self-harm method among all CALD/non-CALD groups. Poisoning by other substances was more common among those from Southern and Central Asia and Sub-Saharan Africa (13–14%), and self-injury by sharp object was more common among those from North East Asia (15.4%) compared to other groups (Table 1).

### Outcomes following self-harm in CALD versus non-CALD groups

#### In-hospital death

Among all self-harm hospital inpatients *(including those who died in hospital and within 12-months of the index hospital discharge)*, in-hospital death during POC occurred in 1.0% of those from CALD backgrounds, and 0.7% of those from the non-CALD group. It was most common in those originally from North-West Europe (2.1%). Fully adjusted logistic regression modelling (adjusted for demographic characteristics, injury-related variables and pre-existing health service use) demonstrated that the odds of in-hospital death were *lower* in CALD than in the non-CALD group (Table 3).

#### Readmission due to self-harm

Self-harm readmission occurred in 12.9% of self-harm patients (excluding *those who died in hospital and within 12-months of the index hospital discharge)*. The prevalence was lower in CALD people (8.1%) than the non-CALD counterparts (13.6%) (*p* < 0.001). Having at least one readmission due to self-harm was least common among those from Asian backgrounds (5.2–7.1%).

Table 3 shows the results of the ZINB models to compare the risk of readmissions due to self-harm in CALD groups vs the non-CALD group. The overall patterns and trends were similar between the univariable and multivariable ZINB models. The logistic model component of the ZINB model showed the odds of having zero repeat self-harm hospital admission did not differ between the two groups. However, the negative binomial component showed among those who were readmitted to the hospital due to self-harm, patients in the CALD group had 20% fewer readmissions than the non-CALD group.

By regions of birth, the count component showed that among those who had at least one self-harm readmission, those from Southern and Central Asia had 40% fewer hospital readmissions than non-CALD people.

#### Subsequent ED presentation due to self-harm

One in five self-harm inpatients presented at the ED with another self-harm, the prevalence was also less common in the CALD group (14.8%) compared to the non-CALD group (20.9%).

The ZINB model showed that among patients who had at least one subsequent visit to the ED due to self-harm, those from CALD backgrounds had 10% fewer presentations than the non-CALD group. Within specific ROB, those with North African and Middle Eastern backgrounds had 30% fewer and Southern and Central Asian backgrounds had 20% fewer additional visits. No statistically significant differences were found in the logistic regression components.

The results *of readmission due to injury; repeat ED presentation due to injury were similar* to self-harm. Those from Asian backgrounds had the lowest proportions of readmission due to injury (Appendix 2).

#### Clinical mental health contacts

Clinical mental health service contacts following self-harm were made in 63.6% of patients, with CALD people (53.8%) less likely to make contact than the non-CALD group (65.1%, *p* < 0.001) (Table 2).

The count component showed that among those who contacted mental health services at least once, the CALD group had 10% fewer contacts than the non-CALD group. By regions of birth (ROB), those from South-East Asia, Southern and Central Asia and Southern and Eastern Europe had 10–20% fewer contacts, but those from North-East Asia and Sub-Saharan Africa had 20–30% more contacts than the non-CALD. The logistic component also found 40% higher odds of not visiting mental health after self-harm among some CALD people compared to the non-CALD group (Table 3).

#### Immortal time bias

The approach of excluding those who died within 12 months of self-harm hospital discharge from the analysis of health services use after self-harm may have led to ‘immortal time bias’ (Ferrie and Ebrahim, 2014). People with a higher risk of repeat self-harm and suicide may have been excluded because the risk of repeat self-harm suicide following self-harm is highest within 12 months after self-harm (Geulayov et al., 2019). To address this potential bias, we compared deaths in all groups and found no difference in the likelihood of death within 12 months among nine CALD groups by ROB and the non-CALD group, but it was statistically lower in the overall CALD group versus the non-CALD group. This suggests that the removal of 12 month deaths did not affect the finding of differences between ROB and the non-CALD group. However, it may have led to an underestimation of the differences between the general CALD group and the non-CALD group. In other words, the lower risk of repeated hospital visits due to self-harm and lower uptake of mental health services after self-harm admission among study participants with CALD versus non-CALD backgrounds may be more pronounced than reported in this study.

## Discussion

The present study examined the association between cultural background and self-harm-related health outcomes including repeat hospital treatment due to self-harm, mental health service contacts after self-harm and in-hospital death during index self-harm. The results suggest that CALD backgrounds were negatively associated with in-hospital death, repeat self-harm and mental health service use after self-harm. Specifically, while mental health contacts following self-harm offer opportunities for secondary prevention, the results show CALD patients (born in Asia) were less likely to utilise mental health services after self-harm compared to the non-CALD populations. However, CALD people, especially those from Southern and Central Asia, made less use of hospital services due to self-harm readmissions and repeat ED presentations (among those who had at least one hospital revisit with self-harm). The findings of this study and other work suggest a complex interplay between cultural background and health-related outcomes of self-harm (Borrill et al., 2011; Cooper et al., 2006; Pham et al., 2023).

### Repeat hospital-treated self-harm

One in 10 self-harm inpatients had additional hospital admissions arising from the first self-harm admission within a 12 months period, and 2 in 10 had additional ED presentations with self-harm. Of those who had additional admissions/ED presentations, CALD people (from Asia) made less use of hospital services due to repeat self-harm than non-CALD people. The same pattern was also found when comparing the rate of repeat self-harm in Australia to rates in other birth countries (for which repeat self-harm statistics were available). Specifically, the rate was higher than in Taiwan (8%) (Chung et al., 2012) and Sri Lanka (3.1%) (Knipe et al., 2019); however, slightly lower than in the United Kingdom (Hawton et al., 2012; Owens et al., 2002). It is noteworthy that comparisons are difficult to make because of differences in data collection and study methodology, and CALD groups in Australia may not be (statistically) representative of the population in their birth country (due to for e.g. the healthy migrant effect). However, the pattern suggests the possibility that migrants might carry their risk and protective factors for self-harm from their birthplaces to the migrated countries. Future research is recommended to test this possibility.

The overall incidence of self-harm and health service use observed here and elsewhere (Pham et al., 2023) may underestimate the true extent of the problem. People who engaged in self-harm may be less likely to seek help from services following self-harm, especially those from CALD backgrounds. They may also be more likely to report self-harm as an unintentional injury (El Halabi et al., 2020) due to specific socio-cultural factors (e.g. shame, religious beliefs/mores and self-harm or mental health-related stigma). Notwithstanding these possible underestimations, the finding is consistent with U.K. studies (Borrill et al., 2011; Cooper et al., 2010) where White people were more likely to report repeated self-harm than those from ethnic minority groups. However, these studies might have suffered from similar limitations. Therefore, there is a need for prospective cohort studies in CALD communities that seek a broader sociocultural context and that can longitudinally study people who have engaged in self-harm regardless of whether they were admitted to hospitals.

Moreover, as self-harm might be (mis)reported as other injuries (El Halabi et al., 2020) due to the sociocultural factors discussed above, we might expect higher rates of injury in CALD groups. To examine this possibility we also tested the relationship between CALD backgrounds and hospital-treated injuries (after the index self-harm episode). Our analysis again showed that CALD status was associated with fewer revisits to hospital with injury. Accordingly, it is worth tentatively considering if a CALD background is negatively associated with multiple repeats of (hospital-treated) self-harm. However, the results should be interpreted with caution as we focus on self-harm hospital-admitted patients. Future research is clearly needed to better understand the sociocultural factors underlying this observation (not limited to hospital context), and whether it holds for CALD people arriving from countries with political instability and conflict and associated psychological trauma.

### Mental health contacts after self-harm

An important outcome of interest in the present study was mental health service contact following self-harm. Although hospital presentation for self-harm is an opportunity for psychological management and other self-harm interventions (Larkin and Beautrais, 2010), only around 50% of CALD and non-CALD patients, who had not previously used clinical mental health services before self-harm, used them after self-harm. The *utilisation of mental health services* after self-harm was lowest among Asian-born people. There are different ways to interpret this. Asian-born people might have a lower prevalence of mental health illnesses and less need to access services. Results of a systematic review found that South Asian adults engaged in self-harm impulsively in response to life events rather than in association with a psychiatric illness (Bhui et al., 2007). In contrast, lower uptake of health services might be due to stigma, systemic racism in service provision, shame around mental health and self-harm (Kramer et al., 2002) and poor awareness of mental health and available services or the use of alternative medicine among some CALD groups (Sue et al., 2012). The study data were unsuited for testing these possible explanations and whether the relative under-utilisation of mental health services was due to a lower need for mental health services or cultural barriers as discussed above. Future research is needed to explore these possibilities and self-harm intervention should consider them to maximise its effectiveness.

### In-hospital death

The final outcome of interest was in-hospital death during the first self-harm period of care. To our knowledge, this outcome had not been examined in previous work, despite it being a significant endpoint and important for prevention efforts. The proportion of index admissions resulting in-hospital death was just under one per cent. Notably, after accounting for relevant factors, the analysis showed that CALD people were less likely to die in hospital than non-CALD populations. It is worth noting that many people who have self-harmed may have died before they could make it to hospital; this study does not address potential CALD/non-CALD differences in ‘pre-hospital death’.

### Limitations

The study has a few limitations. First, due to the unavailability of the Mental Health Community Support Services dataset within the time frame, we did not examine non-clinical mental health service contacts as an outcome of self-harm. Second, there are also well-known limitations in using country of birth only to define CALD backgrounds as discussed elsewhere (Pham et al., 2023). As most Indigenous peoples in Victoria were born in Australia or English-speaking countries, they were mostly included in the non-CALD population (which is based on country of birth). This may have increased the incidence of self-harm in the non-CALD group because the rates of self-harm in Indigenous peoples have been reported to be relatively high (AIHW, 2022). Next, comprehensive data on whether people in CALD groups arrived in Australia through refugee status was unavailable, and this factor should be examined when the data is available. Finally, the findings might also be influenced by the length of stay in Australia of migrants and acculturation, as higher levels of acculturation appear to increase the risks of lifetime self-harm (Perez-Rodriguez et al., 2014).

### Implications

Repeat self-harm was common among those with history of self-harm: 1 in 10 self-harm hospital inpatients, and 1 in 5 ED presentations repeats self-harm in the next year. Among those with at least one repeat, CALD people were less likely to have multiple repeat self-harm than the non-CALD.

CALD people also made less use of clinical mental health services after self-harm. They may be avoiding reporting self-harm to health practitioners in different settings as well as avoiding the use of mental health services because of underlying cultural factors. Understanding whether and how sociocultural background may affect both the reporting of self-harm and mental health service access prior to and subsequent to self-harm in CALD groups could inform clinical practices to prevent the recurrence of self-harm and better recognition of self-harm in CALD groups.

## Appendix 1

### Data sources

Several databases were used within the data linkage study.

1. Victorian Admitted Episodes Dataset (VAED): Hospital admission cases were extracted from the VAED, which records all hospital admissions in public and private hospitals in the state of Victoria.
2. Victorian Emergency Minimum Dataset (VEMD): Emergency department (ED) presentation cases were extracted from VEMD, which records ED presentations at 39 Victorian public hospitals with 24-hour emergency departments.
3. Clinical public mental health services (CMI/ODS): Clinical services focus on the assessment and treatment of people with a mental illness. These services are called area mental health services and are managed by general health facilities, such as hospital.
4. Victorian Death Index (VDI): derived from Births, Deaths and Marriages data. The VDI was used as an indicator of death due to any cause, for data exclusion in evaluating repeat self-harm and mental health contact within 12-month hospital discharge.

### Data linkage process

Hospital inpatient data (from VAED) was used for sample selection and to determine outcomes. Outcomes were determined by following the cohort over 12 months using linked hospital data (VAED and VEMD) and Clinical public mental health services data (CMI/ODS) (Figure 1). A look-back period of 12 months before the index event was used to identify pre-existing self-harm and mental illnesses (evidenced in hospital and clinical mental health contact records) (Figure 2).

## Appendix 2

### Associations between CALD status and readmission and ED presentation due to injury 1 year following index self-harm hospital admission

#### Readmission due to injury

Similar to readmission due to self-harm, readmission due to injury was less common in CALD groups (13.6%, ranging from 1 to 11 admissions in 12 months) compared to the non-CALD group (19.1% ranging from 1 to 31). Those from Asian backgrounds had the lowest proportions of readmission due to injury.

The count component of the ZINB model showed that among patients who were readmitted to hospital due to injury following the index self-harm event, those from CALD groups had 10% fewer readmissions due to injury than the non-CALD group. Specifically, the ZINB model comparing specific ROB with the non-CALD group found that patients from North Africa and the Middle East; South-East Asia, Southern and Central Asia and Sub-Saharan Africa had 30–40% fewer readmissions to hospital due to injury. The Zero model of the ZINB model found that those from the Americas were 5.3 times less likely to be readmitted to hospital with injury than the non-CALD.

#### Subsequent ED presentation due to injury

Subsequent ED presentation due to injury was also less common in the CALD group (28.3%, ranging from 1 to 15 presentations) compared to the non-CALD (38.6%, ranging from 1 to 88 presentations in 1 year). Subsequent presentations due to self-harm were least common in those from Asian regions of birth (Table 2).

The ZINB model showed that among patients who subsequently visited the ED due to injury, those from CALD backgrounds had 20% fewer presentations to the ED with injury than the non-CALD group; within specific ROB, the percentages were 30% less likely for those from North Africa and the Middle East, and 20% for those from Southern and Central Asia (Appendix 2-Table 4).

**Table 4.** Associations between CALD status and hospital service use due to injury 1-year following index self-harm hospital admission. Reference group: Non-CALD.

	Readmission due to injury						ED presentation due to injury					
	IRR	*95% CI*		OR	*95% CI*		IRR	*95% CI*		OR	*95% CI*	
**Univariable models**												
**CALD status**	**0.8** †	***0.7***	***0.8***	**1.0**	***0.8***	***1.2***	**0.6** †	***0.5***	***0.6***	**0.4** †	***0.3***	***0.7***
**CALD groups by regions of birth**												
Oceania and Antarctica*	0.8	*0.4*	*1.5*	0.6	*0.2*	*2.0*	0.6†	*0.4*	*0.9*	0.2	*0.0*	*4.1*
North-West Europe*	0.8	*0.6*	*1.1*	0.6	*0.3*	*1.2*	0.8†	*0.6*	*0.9*	0.5	*0.2*	*1.0*
Southern and Eastern Europe*	0.7†	*0.5*	*0.9*	0.7	*0.4*	*1.1*	0.6†	*0.5*	*0.7*	0.4†	*0.2*	*0.7*
North Africa and the Middle East	0.7†	*0.5*	*1.0*	0.9	*0.5*	*1.6*	0.6†	*0.5*	*0.8*	0.9	*0.4*	*1.8*
South-East Asia	0.6†	*0.4*	*0.9*	1.2	*0.7*	*2.1*	0.5†	*0.4*	*0.6*	0.8	*0.4*	*1.8*
North-East Asia	0.8	*0.5*	*1.4*	1.9	*1.0*	*3.7*	0.7†	*0.5*	*0.9*	3.5†	*1.7*	*7.3*
Southern and Central Asia	0.5†	*0.3*	*0.7*	1.1	*0.6*	*1.9*	0.4†	*0.4*	*0.5*	0.3	*0.1*	*1.1*
Americas*	1.4	*0.8*	*2.4*	2.1	*1.0*	*4.6*	0.8†	*0.6*	*1.2*	0.9	*0.3*	*3.1*
Sub-Saharan Africa*	0.3†	*0.1*	*0.6*	0.5	*0.1*	*2.5*	0.5†	*0.3*	*0.6*	0.5	*0.1*	*2.6*
**Multivariable models**												
**CALD status**	**0.9** †	***0.8***	***0.9***	**1.1**	***0.9***	***1.4***	**0.8** †	***0.7***	***0.8***	**1.3**	***1.0***	***1.6***
**CALD groups by regions of birth**												
Oceania and Antarctica*	1.1	*0.7*	*1.8*	1.6	*0.4*	*7.2*	0.7	*0.5*	*1.1*	0.6	*0.1*	*3.8*
North-West Europe*	1.1	*0.9*	*1.4*	0.9	*0.2*	*3.9*	0.9	*0.7*	*1.2*	0.8	*0.4*	*1.7*
Southern and Eastern Europe*	1.0	*0.8*	*1.2*	1.1	*0.4*	*3.2*	1.0	*0.9*	*1.2*	1.2	*0.8*	*1.8*
North Africa and the Middle East	0.7†	*0.6*	*1.0*	0.3	*0.0*	*3.0*	0.7	*0.6*	*0.9*	1.3	*0.7*	*2.4*
South-East Asia	0.7†	*0.6*	*1.0*	0.9	*0.3*	*2.6*	0.9	*0.7*	*1.0*	1.8†	*1.1*	*3.1*
North-East Asia	0.9	*0.6*	*1.3*	1.8	*0.6*	*5.3*	1.0	*0.7*	*1.3*	2.1	*1.0*	*4.2*
Southern and Central Asia*	0.7†	*0.6*	*1.0*	1.5	*0.6*	*3.3*	0.8	*0.7*	*1.0*	1.3	*0.7*	*2.5*
Americas*	1.4	*0.9*	*2.2*	5.3†	*1.7*	*16.8*	1.1	*0.8*	*1.6*	3.1†	*1.3*	*7.6*
Sub-Saharan Africa*	0.6†	*0.3*	*1.0*	0.8	*0.1*	*9.0*	0.8	*0.5*	*1.1*	0.5	*0.1*	*3.1*

CALD: Culturally and Linguistically Diverse; ED: emergency department; OR: odds ratio; CI: confidence interval; IRR: Incident Risk Ratio; ROB: region of birth.

* Persons who were born in English-speaking countries were excluded from the relevant CALD groups by ROB, and they were included in the non-CALD group.

† : *p* < 0.05.
